# Potential molecular communication of blood and synovium through ligand-receptor interactions in osteoarthritis

**DOI:** 10.3389/fmed.2026.1719995

**Published:** 2026-01-23

**Authors:** Maochun Wang, Guihua Tan, Shiqi Wang, Wenxue Lv

**Affiliations:** 1 Department of Plastic Surgery, Affiliated Friendship Plastic Surgery Hospital of Nanjing Medical University, Nanjing, Jiangsu, China; 2 Division of Sports Medicine and Adult Reconstructive Surgery, Department of Orthopedic Surgery, Nanjing Drum Tower Hospital, School of Life Sciences, Nanjing University, Nanjing, Jiangsu, China; 3 Division of Sports Medicine and Adult Reconstructive Surgery, Department of Orthopedic Surgery, Nanjing Drum Tower Hospital Clinical College of Nanjing University of Chinese Medicine, Nanjing, Jiangsu, China; 4 Department of Orthopedics, Affiliated Hospital of Shandong University of Traditional Chinese Medicine, Jinan, Shandong, China

**Keywords:** blood, ligand-receptor interactions, osteoarthritis, synovium, transcriptome

## Abstract

**Introduction:**

Osteoarthritis (OA) is a common degenerative joint disease resulting from the breakdown of multiple joint tissues, remains a leading cause of disability with limited therapeutic options. Synovitis is one of the reasons of OA progression, while communication between blood and synovium during disease process is still unclear.

**Methods:**

We used transcriptomic datasets from blood and synovium of healthy controls and OA patients to investigate potential molecular crosstalk between blood and synovium in OA pathogenesis through ligand-receptor pairs.

**Results:**

Ligand-receptor pair analysis revealed 129 ligands and 137 receptors differentially expressed in blood, and 108 ligands and 86 receptors in synovium. Gene ontology enrichment analysis of differentially expressed ligands indicated receptor ligand activity in both tissues, with blood enriched in leukocyte migration, cell chemotaxis, and leukocyte chemotaxis, and synovium in negative regulation of response to external stimulus, epithelial cell proliferation, and cell chemotaxis. Further protein-protein interaction (PPI) network analysis showed that blood ligands were mainly associated with inflammation and immunity (IL6, IL1B, IL23A, IFNA1, and TNF), while several synovium ligands were linked to angiogenesis (TGFB1, FGF7, and PDGFA). Based on ligand-receptor interactions and PPI network of differentially expressed ligands, we predicted and constructed molecular communication map between blood and synovium. Immunofluorescence staining of synovium showed more blood micro-vessels in OA patients and elevated IL6 and IL1B expression levels, suggesting that synovial inflammation might partly originate from pro-inflammatory cytokines in blood.

**Discussion:**

These findings offered new understanding of the molecular mechanisms underlying blood and synovium communication in OA, and provided potential therapeutic drug targets for OA treatment to simultaneously modulate systemic inflammation and local angiogenesis.

## Introduction

Osteoarthritis (OA) is a degenerative joint disease, which is becoming one of the most leading causes of disability (1). OA affects multiple tissues in patients, including cartilage, synovium, meniscus and subchondral bone (2). The patients suffer from pain, stiffness and swelling, while the current treatments can only alleviate the progression of the disease (3). The most common pharmacological treatment is non-steroidal anti-inflammatory drugs (NSAIDs) (4). These agents mainly target the inflammatory mediators IL-1 and COX-2 to achieve analgesic and anti-inflammatory effects (5, 6). However, long-term use requires vigilance for gastrointestinal and cardiovascular side effects (7). Once the disease reaches end stage, it can only be treated through surgical replacement (8).

Synovitis is one of the major factors promoting the progression of OA, manifested as swelling of the joint accompanied by thickening of the synovial membrane and vascular proliferation (9). It is known that synovial inflammation is mainly stimulated by the breakdown of articular cartilage and subchondral bone during OA (9–11). This may result in a self-regulatory inflammatory response, which triggers synovium to release inflammatory mediators and aggravates the effects of inflammation (11). A lot of inflammatory mediators can promote synovial inflammation, of which interleukin-1β (IL-1β) and tumor necrosis factor *α* (TNFα) are the main proinflammatory cytokines involved in the progression of OA (12). They can not only mediate their own expression, but also induce the generation of matrix metalloproteinases (MMPs) and other cytokines (e.g., IL6, IL8, PTGS2), which lead to articular cartilage damage and further aggravate OA progression (13). Notably, the degree of synovial angiogenesis is associated with the grade of synovitis in OA (14). Some inflammatory markers are able to be detected in the circulation system, which raises the concern of inflammatory mediators derived from blood (15). The angiogenesis in the synovium is another significant feature of inflammatory synovium, which is due to the imbalance of proangiogenic and antiangiogenic factors (16). Macrophages within synovium may secrete cytokines that induce angiogenesis or stimulate endothelial cells and fibroblasts to produce angiogenic factors, such as vascular endothelial growth factor (VEGF) (17, 18). VEGF promotes synovial angiogenesis through highly expressed VEGF receptor 2 (VEGFR2) in endothelial and synoviocytes, which result in the emerge of micro-vessels and pannus (19–21). However, the mechanism of the communication between synovium and blood in the physiological and pathological osteoarthritis is still unclear.

Here, we utilized the transcriptome of blood and synovium samples in healthy and osteoarthritis patients to identify differential expressed genes, and further screening of ligand-receptor pairs within two tissues to unveil the communication between blood and synovium in osteoarthritis. We constructed molecular networks that may reveal the role of synovium in angiogenesis and the pro-inflammatory role of blood on synovium. Further immunofluorescence detection revealed elevated IL6 and IL1B levels in blood micro-vessels. These results may provide new insights into future therapeutic drug targets.

## Materials and methods

### Data sources and quality control

Transcriptome of blood and synovium samples in osteoarthritis patients were acquired from gene expression omnibus (GEO) database, including samples derived from peripheral blood mononuclear cells (PBMCs, GSE48556, HC = 33, OA = 108) (22) and synovial fibroblasts (SFs, GSE29746, HC = 11, OA = 11) (23). All raw data were normalized by robust multiarray average (RMA) algorithm in RStudio (2023.06.2 + 561), probe IDs were transformed to gene symbols and the mean values were calculated as the expression values. There were 25,159 and 19,749 unique genes obtained from PBMC and SF, respectively. Principal component analysis (PCA) was performed by FactoMineR (v2.8) and factoextra (v1.0.7) with R packages.

### Analysis of blood and synovium transcriptome

Differential expressed genes (DEGs) of two datasets were filtered by *p* value, which was less than 0.05. DEG were shown on volcano plot with ggplot2 (v3.4.2), of which the most 20 statistical differences DEG were labeled with gene symbols. To illustrate the communication between two tissues, ligands and the corresponding receptors within DEG were filtered by ligand-receptor database based on the FANTOM5 project. Ligand-receptor pairs within and between PBMC and SF were plotted by RCircos (v1.2.1). Differential expressed ligands were enriched by gene ontology (GO), including molecular function, cellular component and biological process. Then, the potential ligand interaction network was analyzed in STRING and constructed by cytoscape (v3.9.0). Heatmap was constructed by pheatmap (v1.0.12).

### Ethical statement and immunofluorescence staining

Human synovium samples were obtained from two patients with OA undergoing total knee replacement surgery and one patient involved in a traffic accident requiring amputation. The use of human samples was approved by Ethical Committee of Affiliated Hospital of Shandong University of Traditional Chinese Medicine (AF/SC-08/03.0), and the patients provided written informed consent to participate in this study.

The synovium samples were serially sectioned into paraffin slices of 5 μm thickness. After deparaffinization and dehydration, they were blocked with 5% BSA. Primary antibodies against IL6 (Proteintech, China) (24) and IL1B (Proteintech, China) (25) were applied and incubated overnight at 4 °C. After washing with PBST, the corresponding fluorescent secondary antibodies were applied and incubated at room temperature for 1 h. Finally, the slices were counterstained with DAPI (Abcam, UK) and observed under a fluorescence microscope (Zeiss, Germany).

## Results

### Quality control of blood and synovium transcriptomes

**Figure 1:**
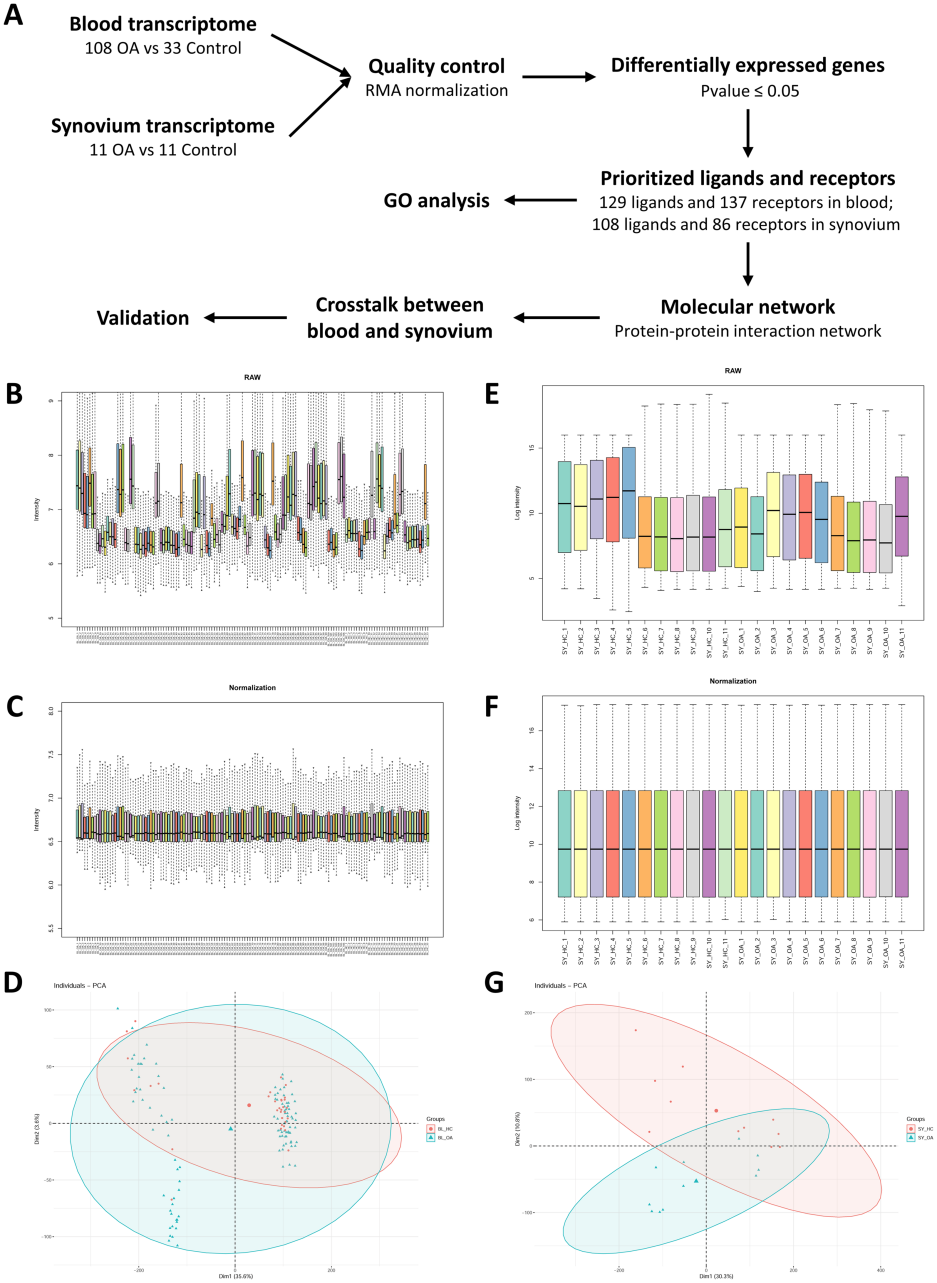
Schematic diagram and quality control of transcriptomic data. **(A)** Schematic diagram summarizing the process of this study. **(B,C)** Box plots of the original **(B)** and normalized **(C)** blood transcriptomic data. **(D)** Principal component analysis of blood transcriptomic data. **(E,F)** Box plots of the original **(E)** and normalized **(F)** synovium transcriptomic data. **(G)** Principal component analysis of synovium transcriptomic data.

Our strategy was using the transcriptomes of blood and synovium tissues from normal controls and patients with osteoarthritis (OA) to identify differentially expressed ligands and receptors. We then predicted molecular communications between synovium and blood tissues based on their ligand-receptor pair interactions (Figure 1A). After downloading the raw data, box plots revealed the differences between the original transcriptomes of blood and synovium tissues (Figures 1B,E). Following normalization, the data showed consistency, with the medians of different datasets in consistent positions (Figures 1C,F), especially for the synovium transcriptome (Figure 1F). Principal component analysis (PCA) showed that in the blood transcriptome, the normal group was more dispersed, while the OA group was relatively concentrated (Figure 1D). In contrast, in the synovium transcriptome, both the normal and OA groups showed relatively tight clustering (Figure 1G).

### Screening of differentially expressed genes and ligand-receptor pair analysis

**Figure 2:**
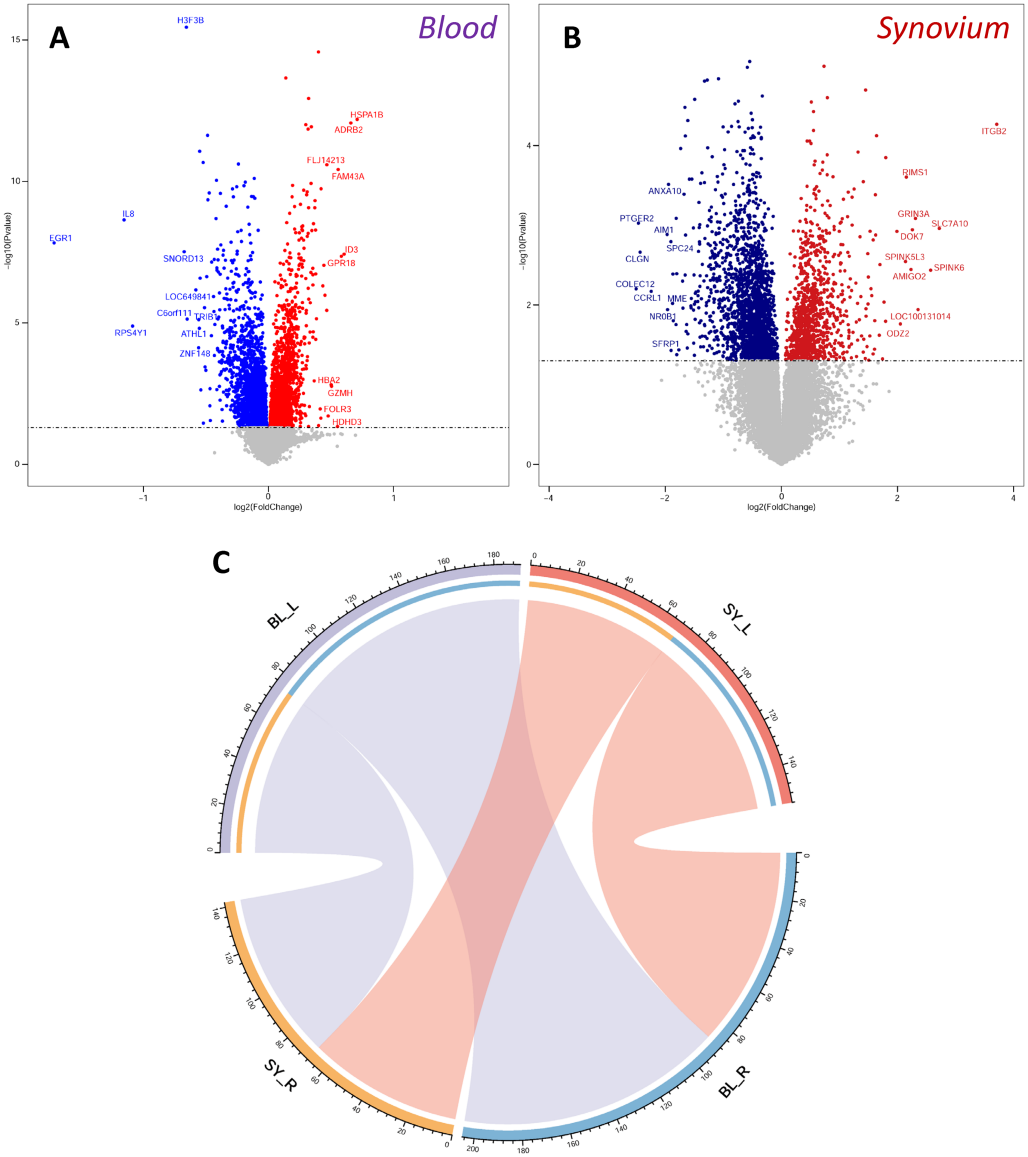
Differentially expressed genes and ligand-receptor interaction analysis. **(A,B)** Volcano plots of differentially expressed genes in blood **(A)** and synovium **(B)** transcriptomes. Red indicates up-regulated genes, and blue indicates down-regulated genes. The top 10 up-regulated and down-regulated genes are labeled. **(C)** Analysis of ligand-receptor interactions between blood and synovium. BL, blood; SY, synovium.

We screened differentially expressed genes using a unified threshold of *p* < 0.05. In the OA blood transcriptome, genes such as HSPA1B, ADRB2, FLJ14213, FAM43A, and ID3 were significantly upregulated, while H3F3B, IL8, EGR1, SNORD13, and LOC649841 were significantly downregulated (Figure 2A). In the OA synovium transcriptome, ITGB2, RIMS1, GRIN3A, SLC7A10, and DOK7 were significantly upregulated, whereas ANXA10, PTGER2, AIM1, SPC24, and CLGN were significantly downregulated (Figure 2B). Many differentially expressed genes were consistent with the results of original dataset analysis (22, 23), demonstrating the availability and accuracy of the datasets. Further analysis using a ligand-receptor database identified differentially expressed ligands and receptors. We found 129 ligands and 137 receptors differentially expressed in the blood, and 108 ligands and 86 receptors differentially expressed in the synovium. Through ligand-receptor interactions, we predicted molecular communications between blood and synovium (Figure 2C). In addition to interactions within blood and synovium, there were also many potential molecular communications between these two tissues.

### Gene ontology analysis and molecular network construction

**Figure 3:**
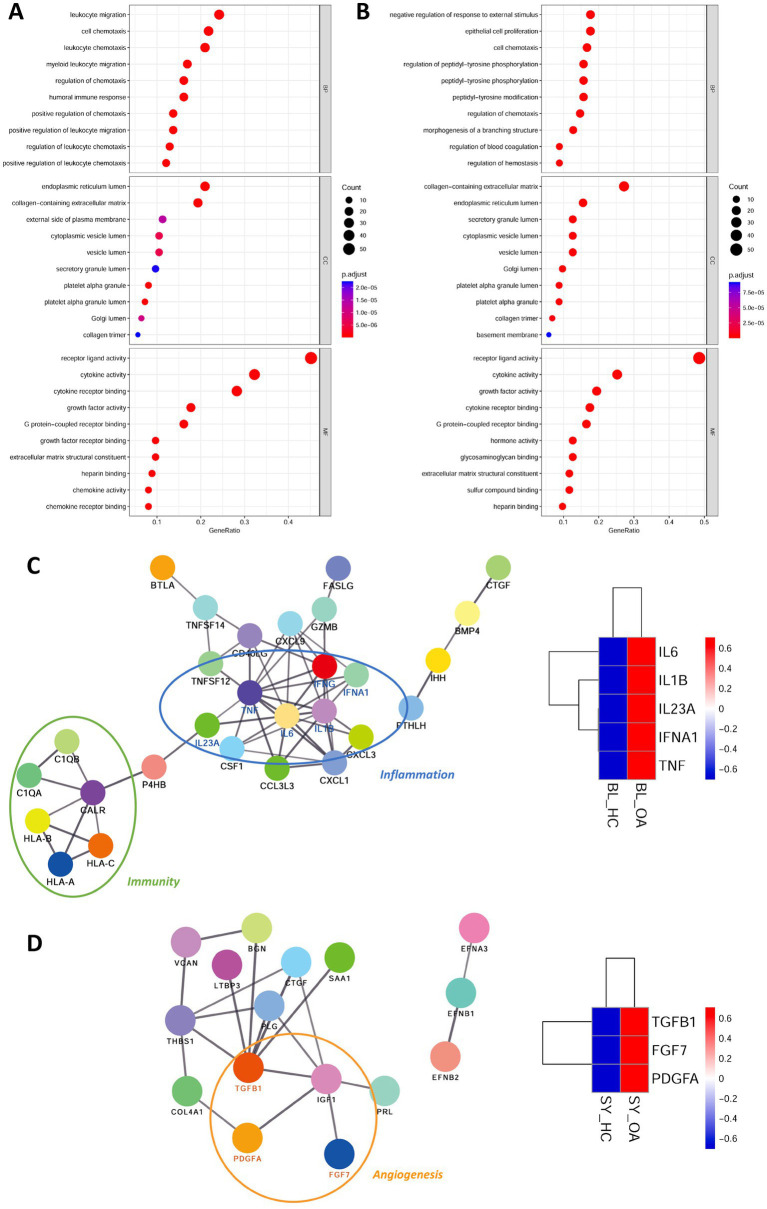
Gene ontology (GO) analysis of differentially expressed ligands and construction of PPI (protein–protein interaction) molecular networks. **(A,B)** Dot plots showing GO terms of differentially expressed ligands in blood **(A)** and synovium **(B)** transcriptomes. **(C,D)** PPI molecular networks of differentially expressed ligands in blood **(C)** and synovium **(D)** transcriptomes. The schematic on the right shows the heat map of IL6, IL1B, IL23A, IFNA1, and TNF expression in blood, as well as TGFB1, FGF7, and PDGFA in synovium. BL, blood; SY, synovium; OA, osteoarthritis.

We performed gene ontology enrichment on the differentially expressed ligands. In terms of molecular function, both blood and synovium tissues were primarily enriched in receptor ligand activity, indicating the accuracy of our ligand screening (Figures 3A,B). In terms of cellular components, they both showed enrichment in endoplasmic reticulum lumen and collagen-containing extracellular matrix (Figures 3A,B). In biological processes, the blood transcriptome was mainly enriched in leukocyte migration, cell chemotaxis, and leukocyte chemotaxis (Figure 3A), while the synovium transcriptome was primarily enriched in negative regulation of response to external stimulus, epithelial cell proliferation, and cell chemotaxis (Figure 3B). This highlighted the functional differences of differentially expressed ligands between blood and synovium tissues. We then imported these differentially expressed ligands into the STRING database to construct a protein–protein interaction (PPI) molecular network. The molecular network of blood ligands was mainly associated with inflammation and immunity, with IL6, IL1B, IL23A, IFNA1, and TNF being upregulated in the blood of OA patients (Figure 3C). In the molecular network of synovium ligands, angiogenesis-related pathways were observed, with TGFB1, FGF7, and PDGFA being upregulated in the synovium tissue of OA patients (Figure 3D).

### Prediction and validation of synovium-blood interaction molecular network

**Figure 4:**
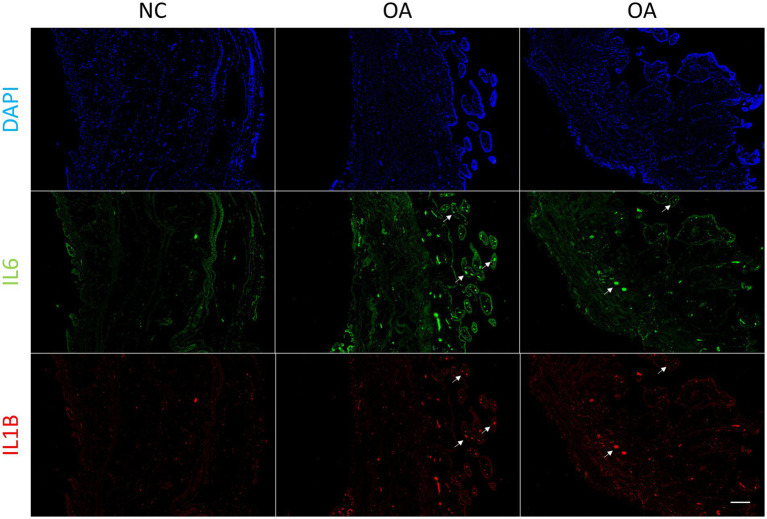
Immunofluorescence of synovium tissue. Immunofluorescence of IL6 and IL1B in normal and OA synovium tissues. White arrows indicate the locations of some blood micro-vessels in the synovium. Human synovium samples were obtained from two patients with OA and one patient requiring amputation. Scale bars, 200 μm. DAPI, blue; IL6, green; IL1B, red. NC, control; OA, osteoarthritis.

**Figure 5:**
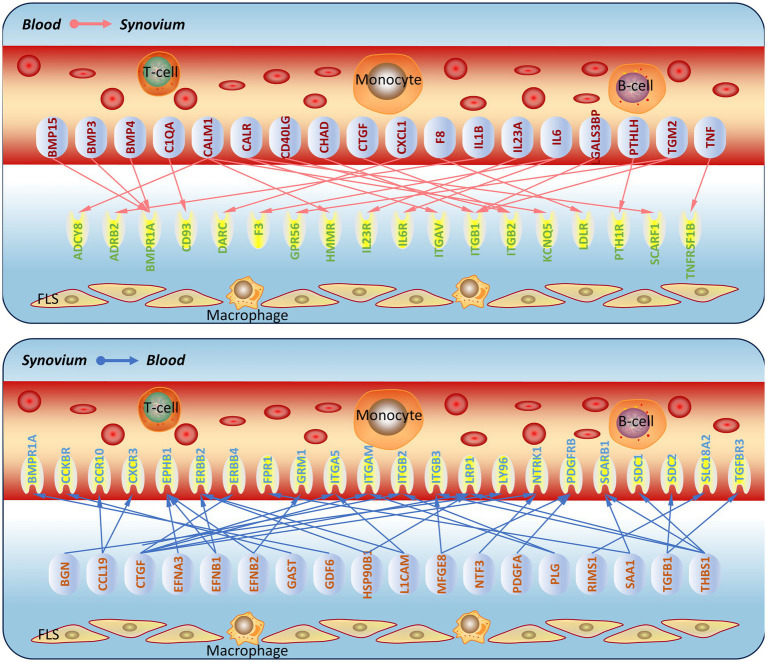
Prediction of molecular communication between blood and synovium in osteoarthritis. Based on the screened ligand molecular network and ligand-receptor interactions, a schematic of molecular communication between blood and synovium in osteoarthritis was constructed.

In order to verify whether synovial inflammation partly originated from blood, we performed immunofluorescence on synovium sections. Using synovium tissues from amputees as the control, control and OA synovium tissues were stained to detect IL6 and IL1B expression. In OA patients, there were more newly-formed micro-vessels, and the expression of IL6 and Il1B increased in OA blood (Figure 4). This indicated that inflammation in OA synovium might partly due to the elevated pro-inflammatory cytokines IL6 and IL1B in blood. Based on PPI molecular network and ligand-receptor interactions, a molecular interaction network between synovium and blood was further constructed (Figure 5). This map could lay the foundation for studying synovium-blood interactions and provide insights for developing drugs to simultaneously control synovial inflammation and neovascularization in OA.

## Discussion

Understanding the complex molecular interactions in OA progression is crucial for developing more effective therapeutic approaches. Synovitis is a key pathological feature of OA, contributing significantly to disease progression, which is accompanied by the formation of micro-vessels and pannus (26, 27). However, the molecular mechanisms underlying whether the newly formed pannus communicates with the synovium to further aggravate the progression of OA or whether synovial inflammation promotes angiogenesis remain unclear. Therefore, elucidating the tissue communication between the synovium and blood will help develop new therapeutic approaches for OA.

The differentially expressed genes in the blood and synovium transcriptomes were consistent with the results that reported in the original dataset. For example, the upregulation of HSPA1B, ADRB2, FAM43A, and ID3 and the downregulation of H3F3B, IL8, EGR1, and SNORD13 in the blood of OA patients (22), and the upregulation of ITGB2, RIMS1, and GRIN3A and the downregulation of ANXA10 in the synovium of OA patients (23), which further demonstrated the accuracy of our analysis results. Secondly, we found that many differentially expressed genes have been reported to play roles in OA. For example, HSPA1B is a member of the heat shock protein 70 family, and the RNA-binding protein ZFP36L1 regulates members of the HSP70 family (HSPA1A and HSPA1B), preventing OA onset by inhibiting chondrocyte apoptosis (28). ADRB2 (β2-adrenergic receptor) has been identified as one of the potential targets in the OA-related gene network (22), and its knockout leads to increased calcified cartilage thickness and subchondral bone remodeling in experimental OA in mice (29). H3F3B is a target of miR-10a-5p, which promotes IL-1β-induced chondrocyte apoptosis by regulating H3F3B (30). ITGB2 is a member of the integrin family, involved in cell adhesion and inflammatory responses, and studies have shown that high expression of ITGB2 in synovial fluid can assist in OA diagnosis and serve as a biomarker for OA severity (31). PTGER2 is the receptor for prostaglandin E2, and inhibition of prostaglandin E2 attenuates aberrant alteration of subchondral bone, articular cartilage degeneration and pain in osteoarthritis (32). Other unreported differentially expressed genes may also play a role in OA, and these findings can serve as a basis for future research.

The enrichment of differentially expressed ligands in the blood and synovium transcriptomes showed both similarities and differences. In terms of molecular function and cellular components, most of the enriched terms were similar, such as endoplasmic reticulum lumen, collagen-containing extracellular matrix, leukocyte migration, cell chemotaxis, and leukocyte chemotaxis. In terms of biological processes, the blood was more associated with the functions of leukocytes and the regulation of immune responses, while the synovium showed more involvement of cell signaling and regulation, as well as immune responses. These results also showed correlation with many previously reported findings. The increased expression of IL1B in peripheral blood leukocytes is associated with OA progression and pain (15). And the activated macrophages in the synovium produce pro-inflammatory mediators and various tissue-degrading enzymes that lead to the destruction of cartilage and subchondral bone, thereby aggravating OA progression (33). Among the other biological processes we identified in the synovium, such as morphogenesis of a branching structure, epithelial cell proliferation, and regulation of blood coagulation, suggested that the synovium might interact with the blood through these processes at the molecular level. Further molecular network construction revealed that the blood’s molecular network contained many molecules related to inflammation and immunity (such as IL6, IL1B), while the synovium molecular network contained many molecules related to angiogenesis (such as TGFB1, FGF7). This suggested that the blood may influence the synovium through inflammation and immunity, while the synovium may affect vascular regeneration through angiogenesis. It was known that IL-6 and IL-1β were pivotal pro-inflammatory cytokines in OA, which induced cartilage destruction and synovial inflammation, thereby accelerating OA progression (34). While TGF-β1, FGF7, and PDGFA modulated cartilage and synovium homeostasis through different mechanisms. Local inhibition of TGF-β1 signaling attenuated cartilage damage and osteophyte formation in mouse osteoarthritis (35). The role of FGF7 in OA was not clear, and the current evidence was related to bone-defect repair, where exogenous FGF7 enhanced bone formation in rat mandible defects (36). And exogenous PDGFA increased the proliferation of synovial mesenchymal stem cells (37), which might contribute to osteoarthritis through synovitis. These findings revealed the distinct molecular profiles of blood and synovium tissues in OA, highlighting the importance of targeting both inflammatory and angiogenic pathways for effective disease management.

This study also has many limitations. Initially, to obtain more robust data, we applied a stricter screening threshold (adj. P. Val < 0.05), which yielded 10 ligands and 24 receptors in blood, and 4 receptors and no ligands in synovium, leaving virtually no interacting pairs for downstream analysis. We therefore adopted *p* < 0.05 as the criterion, which allowed us to identify a larger set of potential tissue communications while still retaining statistical significance. Secondly, we focused on the communication between blood and synovium, but OA was a disease involving the coordinated pathology of multiple tissues, including cartilage, subchondral bone, synovium, meniscus, and ligaments, etc. Thirdly, the datasets we used were based on microarray platforms, which can only provide a macroscopic overview of the overall changes. Single-cell sequencing would offer better resolution and reveal more cell types. Fourthly, the datasets here were focused on PBMCs and SFs, while endothelial cells in the blood or macrophages in the synovium may also be involved. Whether these communications occur through intermediate cells requires more transcriptome data. Fifthly, the molecular communications we analyzed were based on ligand-receptor interactions; other undiscovered interactions may be overlooked, and these interactions need further experimental validation, such as Co-IP and Tarnswell assays. Finally, the differentially expressed genes we analyzed were mainly coding genes, while miRNAs, lncRNAs, and others may also participate in tissue interactions through exosomes. Further sequencing and analysis of miRNAs, lncRNAs, and others were needed.

In conclusion, our study provided novel insights into the molecular mechanisms underlying the communication between blood and synovium tissues in OA. The identification of key ligand-receptor pairs and their associated pathways offered potential therapeutic targets for modulating inflammation and angiogenesis. Future research should focus on validating these results in larger cohorts and exploring the therapeutic potential of targeting these molecular interactions. Additionally, further investigation into the role of systemic inflammation and local angiogenesis in OA progression may reveal new strategies for disease modification and prevention.

## Data Availability

The original contributions presented in the study are included in the article/supplementary material, further inquiries can be directed to the corresponding authors.
